# Standardized, App-Based Disinfection of iPads in a Clinical and Nonclinical Setting: Comparative Analysis

**DOI:** 10.2196/jmir.2643

**Published:** 2013-08-14

**Authors:** Urs-Vito Albrecht, Ute von Jan, Ludwig Sedlacek, Stephanie Groos, Sebastian Suerbaum, Ralf-Peter Vonberg

**Affiliations:** ^1^PL Reichertz Institute for Medical InformaticsHannover Medical SchoolHannoverGermany; ^2^Institute for Medical Microbiology and Hospital EpidemiologyHannover Medical SchoolHannoverGermany; ^3^Institute of Cell Biology in the Center of AnatomyHannover Medical SchoolHannoverGermany

**Keywords:** tablet PC, hygiene, disinfection, hygiene, nosocomial transmission

## Abstract

**Background:**

With the use of highly mobile tools like tablet PCs in clinical settings, an effective disinfection method is a necessity. Since manufacturers do not allow cleaning methods that make use of anything but a dry fleece, other approaches have to be established to ensure patient safety and to minimize risks posed by microbiological contamination.

**Objective:**

The ability of isopropanol wipes to decontaminate iPads was evaluated prospectively in a observer blinded, comparative analysis of devices used in a clinical and a nonclinical setting.

**Methods:**

10 new iPads were randomly deployed to members of the nursing staff of 10 clinical wards, to be used in a clinical setting over a period of 4 weeks. A pre-installed interactive disinfection application (deBac-app, PLRI MedAppLab, Germany) was used on a daily basis. Thereafter, the number and species of remaining microorganisms on the surface of the devices (13 locations; front and back) was evaluated using contact agar plates. Following this, the 10 iPads were disinfected and randomly deployed to medical informatics professionals who also used the devices for 4 weeks but were forbidden to use disinfecting agents. The quality of a single, standardized disinfection process was then determined by a final surface disinfection process of all devices in the infection control laboratory. No personal data were logged with the devices. The evaluation was performed observer blinded with respect to the clinical setting they were deployed in and personnel that used the devices.

**Results:**

We discovered a 2.7-fold (Mann-Whitney U test, *z=*-3.402, *P=*.000670) lower bacterial load on the devices used in the clinical environment that underwent a standardized daily disinfection routine with isopropanol wipes following the instructions provided by “deBac-app”. Under controlled conditions, an average reduction of the mainly Gram-positive normal skin microbiological load of 99.4% (Mann-Whitney U test, *z=*-3.1798, *P=*.001474) for the nonclinical group and 98.1% (Mann-Whitney U test, *z=*3.1808, *P=*.001469) for the clinical group was achieved using one complete disinfecting cycle.

**Conclusions:**

Normal use of tablet PCs leads to a remarkable amount of microbial surface contamination. Standardized surface disinfection with isopropanol wipes as guided by the application significantly reduces this microbial load. When performed regularly, the disinfection process helps with maintaining a low germ count during use. This should reduce the risk of subsequent nosocomial pathogen transmission. Unfortunately, applying a disinfection procedure such as the one we propose may lead to losing the manufacturer’s warranty for the devices; this remains an unsolved issue.

## Introduction

Infections are called nosocomially acquired if they occur during a hospital stay. They have an enormous clinical and economical impact for health care systems [1]. In addition, multidrug resistant pathogens represent an increasing problem in hospitals these days [2]. Besides the hands of health care workers (HCW), contaminated medical devices and surfaces play an important role in the transmission of bacterial pathogens. This necessitates considerable effort for environmental infection control in order to prevent the spread of all kinds of microorganisms between patients [3].

**Figure 1:**
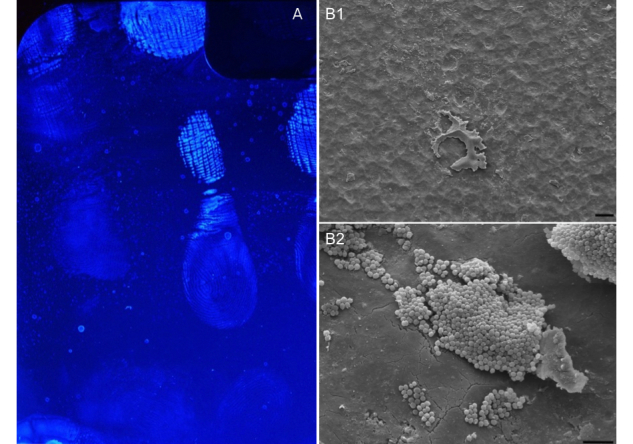
Aluminium backside of a tablet PC with fingerprints and other residue visible under fluorescent light and corresponding scanning electron microscopy pictures of cocci on the device in 2 magnifications (Bars: B1=5µm and B2=10µm).

Mobile devices such as mobile phones or personal digital assistants (PDAs) represent a rather novel “surface” in the hospital setting that may also play an important role in the transmission of nosocomial pathogens. Nowadays, such devices are frequently used by physicians and other medical staff for both clinical practice and educational purposes [4,5]. The number and availability of medical applications (apps) on smartphones is constantly rising and includes drug guides, medical calculators, coding and billing apps, textbooks and other reference materials, classification and treatment algorithms, as well as information regarding general medical knowledge [6]. However, contamination of a device’s surface occurs every time it is being touched by a user [7], and there are several reports showing that these devices may then serve as vectors for transmission of pathogens to patients [8,9]. In a review of data from studies published between 2002 and 2008, Brady et al [10] showed that 9-25% of mobile communication devices were contaminated with pathogenic bacteria. More recent prevalence studies report contamination rates as high as 44-95% [7,11-15]. Like mobile phones, tablet PCs (for example the iPad) are also frequently touched during patient care and bacteria may reside on their surfaces (Figure 1).

Before our study began, tests conducted with microbiological swabs showed that brand new iPad devices are not significantly contaminated with bacteria or fungi. However, the extent of contamination of tablets that have already been used remains yet unknown. Considering that bacteria may survive for days and weeks on inanimate surfaces [16], there is a need to determine the extent of contamination and to implement proper routine decontamination measures.

The present study was set up to determine (1) the microbiological flora (qualitative and quantitative) on tablet PCs as a result of use under the usual conditions that can be found in clinical as well as in nonclinical settings and (2) the quality of a standardized disinfection process as guided by an app specifically programmed for this purpose.

## Methods

### Clinical Setting

A set of 10 Apple iPads was randomly distributed to be used by nursing staff after obtaining informed consent. Various wards of Hannover Medical School, a tertiary care German university hospital, were included. They covered nonsurgical as well as surgical specialties. For the clinical setting, the disinfection study was an add-on to a larger trial dealing with various aspects [17] of using iPads on the wards of the Hannover Medical School. Altogether, approximately 160 staff members on the wards had the opportunity to use the devices. It was not possible to determine how many different individuals had used the iPads. As mentioned in [17], the return rate for questionnaires dealing with the overall project was approximately 26% (42/160); this can be assumed as the minimum of actual individual users. Regarding age and gender, the demographics of the participants who had returned the questionnaire paralleled the values for the nursing staff at the Hannover Medical School, where, at the end of 2012, 83% out of the total 2596 employees of the nursing staff were female (data obtained from the human resources department). For our study, 85% (36/42) were female and the age distribution of 69% (29/42) for those below 45 years of age and 31% (13/42) for those 45 years of age or older was also comparable. It was not possible to determine how they had used the devices and whether all or only some of them had disinfected the devices aided by deBac-app.

**Figure 2:**
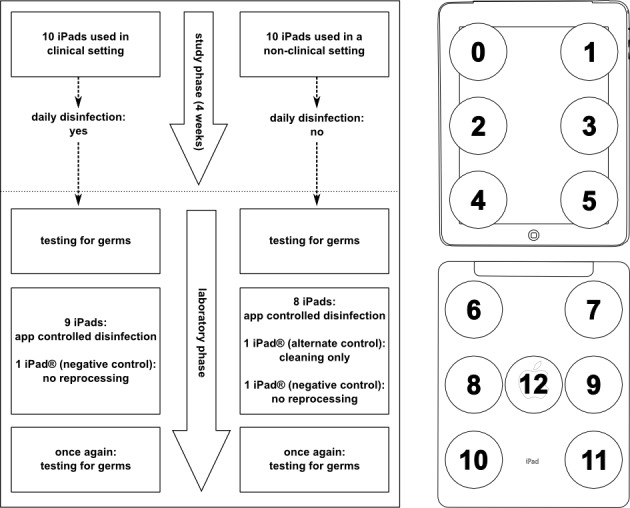
Flowchart on the timeline of the 2 settings of iPad usage (clinical and nonclinical) and contact points used for microbiological sampling of an iPad (surface material was glass on the front side [points 1-6], aluminium [points 7-12], and plastic [point 13] on the backside).

There were no additional accessories such as protective cases, polyurethane foils, rubber or silicone covers, since these may add additional, hard-to-disinfect niches for contaminating pathogens. Instead, we recommended disinfection of the plain surface of every device on a daily basis: once at the beginning of the working shift as well as anytime when an obvious contamination had occurred. Standardization of the disinfection process was achieved by using the “deBac-app” (from the MedAppLab, Hannover, Germany), which was preinstalled on all iPads. This app was designed by our research group and is an interactive cleaning and disinfection guide that is available free from Apple’s App Store [18]. It provides users with simple-to-follow instructions on how to properly disinfect the entire device (Multimedia Appendix 1). Every disinfection process was logged locally on the respective device. No personal data were stored or transmitted to the observers. The study time period of this clinical setting study arm was set to 4 weeks between August 28 and September 19, 2011, in order to achieve a steady state in terms of usage and reprocessing. A flowchart of the study’s timeline is shown in Figure 2.

### Nonclinical Setting

10 iPads were also used for a 4-week time period, between September 23 and October 21, 2011, by 10 members of the medical information technology staff (30% or 3/10 female, aged 23-63 years, mean 41.7, SD 10.6) in the nonclinical study arm. The devices were randomly distributed to the staff after obtaining informed consent for participation in the study. As these staff members generally do not have contact to patients, no regular disinfection of the tablet PCs was performed (Figure 2). All 10 participants of the nonclinical part of the study belonged to the P.L. Reichertz Institute for Medical Informatics at the Hannover Medical School and had volunteered to participate. For the nonclinical setting, there were no dropouts during the course of the study and all participants stated that they had frequently used their devices during the 4-week period.

### Microbiological Testing

After 4 weeks of usage, all devices were examined for growth of microorganisms on their surfaces as soon as they arrived at the microbiological laboratory. Culture media with a contact area of 25 cm^2^ (CASO contact agar plates, Heipha diagnostica Dr. Müller GmbH) were used. These culture media support growth of most aerobic bacteria, molds, and yeasts. Since it is known that the adherence and survival of microorganisms may vary depending upon the type of surfaces material [15], we decided to perform the microbiological sampling for 13 different contact points of each iPad (Figure 2, right), including locations on the front and the back side of the device. These contact points covered all types of material to be found on the surface of the devices (glass, plastic, and aluminium). The contact plates were incubated at 37°C for 18 hours under aerobic conditions. Colony forming units (CFU) were then counted, and species differentiation was carried out in the microbiology laboratory of our facility according to the national guidelines of the German Institute for Standardization DIN EN ISO 15189 as certified by the German Accreditation Council (DAR). The evaluation was conducted in such a way that the laboratory was unaware of the setting to which the iPads had been deployed.

### Electron Microscopy

For photo documentation of bacterial contamination on the tablet PC’s surfaces, scanning electron microscopy (SEM) was applied (Figure 1). Specimens were fixed in 0.1M Na-Cacodylate-HCl buffer (pH 7.3) containing 3% glutaraldehyde for at least 4 hours at 4°C. After washing in the buffer of the fixative, the cells were postfixed in 2% OsO4 buffered in Na-Cacodylate 0.1 M for 90 minutes at room temperature, dehydrated in ascending concentrations of acetone, and subsequently dried in a Balzers CPD 030 critical point dryer (Bal-Tec-AG). After mounting on aluminium stubs with conductive carbon cement (Plano) and sputter coating with gold in a Polaron E 5400 sputter coater, the samples were investigated in a Philips SEM 505 scanning electron microscope at an acceleration voltage of 10kV. Images were recorded using the SEM software version 2.0 [19].

### Final Reprocessing

After primary microbiological testing, the devices underwent final reprocessing performed by laboratory staff (Figure 2). For the clinical setting, with one exception, all devices were disinfected using isopropanol wipes (mikrozid-AF, Schülke & Mayr GmbH) using the 6-step disinfection process guided by deBac-app as it was described above (Multimedia Appendix 1). The remaining 10th iPad did not get reprocessed and, thus, served as a negative control for the disinfection process. For the 10 iPads that had been used in the nonclinical setting, we chose a slightly different approach: 8 of them were disinfected as described, while 1 remained without treatment and 1 was simply cleaned (but not disinfected) by using a new “soft, lint-free cloth”, without any liquid cleaning agents, as recommended in the instructions of the manufacturer of the iPad [20]. A final microbiological testing as described above was performed following the different, aforementioned types of reprocessing performed in the laboratory (Figure 2).

### Nasal Swabs

People may be physiologically colonized by *Staphylococcus aureus* in the anterior nose, and some of these strains even show multidrug resistance, so-called methicillin-resistant *Staphylococcus aureus* (MRSA) [21]. For a comparison of the *Staphylococcus aureus* colonization status of the 10 medical informatics professionals and the surface of their devices, nasal swabs (Transystem, Lot 9275, Hain Lifescience) were taken from the users after informed consent was obtained. Swabs were cultured on Columbia 5% sheep blood agar (Becton, Dickinson) overnight at 37°C. Species identification and susceptibility testing were then performed according to laboratory standard operation protocols.

### Statistical Analysis

We expected a very strong effect of the applied method on reduction of the CFU according to the literature [22]. Therefore, a smaller sample size was expected to be sufficient to demonstrate the efficacy of the disinfection. Calculated for a paired nonparametric test [23], a sample size of 6 iPads per group was considered sufficient to show a significant effect of a reduction of 98% with beta=.20. The sample size calculation was performed with nQuery Advisor V.7, Statistical Solutions. Since a normal distribution of bacteria on the devices and sampled locations could not be confirmed by descriptive statistics, the Wilcoxon signed-rank test and the Mann-Whitney U test were applied (IBM SPSS Statistics Version 20). All tests were performed two-sided, with alpha=.05. Microsoft Excel 2007 was used for qualitative descriptive and quantitative data analysis. Intraclass correlation could not be confirmed following Shrout and Fleiss’ two-way random single measures (consistency) approach computed with SPSS [24].

## Results

### Qualitative and Quantitative Analysis of Microbiological Flora

A total of 6811 colonies representing microbial growth were detected during the initial testing of the iPads after use: 1842 CFU on tablet PCs from the hospital wards where the devices had been disinfected regularly, compared to 4,969 CFU recovered from tablet PCs from the nonclinical setting where daily disinfections had not been carried out (Mann-Whitney U test, *z=*-3.402, *P=*.000670). The distribution of pathogens on the various parts of the tablet PCs is shown in Table 1. Note that most pathogens found in both study arms (clinical and nonclinical) were gram-positive bacteria. A more detailed analysis of the species is shown in Table 2. The majority of microorganisms were members of the physiological microbiota of the human skin. The distribution did not differ significantly between both study arms. The main bacterial genera grown from iPads from the clinical setting were staphylococci (n*=*1104; 59.9%) and micrococci (n*=*469; 25.7%). The same types of bacteria were mainly found on iPads from the nonclinical setting (staphylococci: n*=*3678; 74.2% and micrococci: n*=*1051; 21.2%). However, the cultured microorganisms also included several pathogens. The most frequently identified pathogen was *Staphylococcus aureus* (non-MRSA only), which was found in nasal swabs from 2 medical informatics professionals as well as on their tablet PCs, but this species was also detected on tablet PCs from 2 other staff members who were not colonized themselves (Wilcoxon signed-rank test, *z=*-1.414, *P=*.157).

### Determination of the Quality of the Guided Standardized Disinfection Process

**Figure 3:**
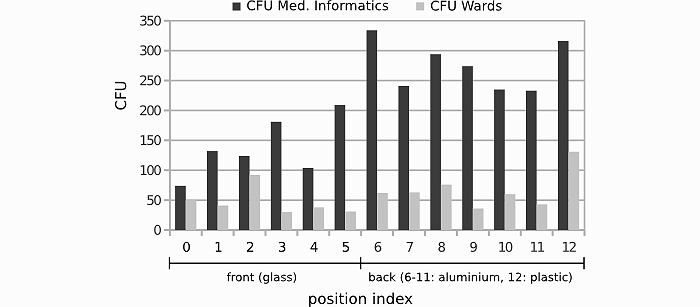
Histogram of CFU-count per localization samples taken from 6 corresponding devices in a clinical and nonclinical setting, stratified for position number, side, and material.

**Figure 4:**
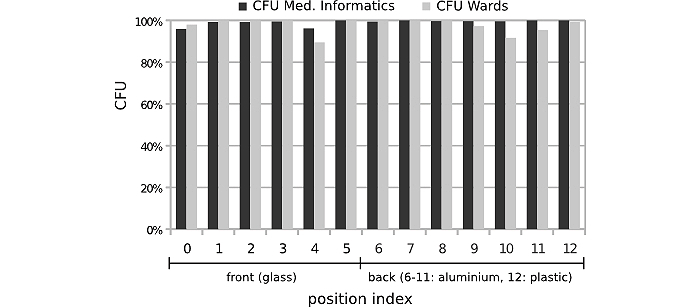
Reduction of CFU in percent per position, side, and material after disinfection.

The percentage of reduction of pathogens on iPads that underwent the standardized disinfection protocol is shown in Table 3 and Figures 3 and 4. A significant overall reduction on microbes was achieved for both the clinical setting (98.1%; Wilcoxon signed-rank test, *z=*-3.1808, *P=*.001469) and the nonclinical setting (99.4%; Mann-Whitney U test, *z=*-3.1798; *P=*.001474). Note that bacilli are capable of forming spores. If doing so, those spores then show an extraordinarily high resistance towards disinfection processes (as they cannot at all be inactivated by alcohol-based disinfection) and other extreme environmental effects [25]. Still, a reduction of *Bacillus* spp. of 88% was achieved in our study in both settings. The reduction rates of all other bacterial and fungal species were as high as 99%.

### Re-Sampling of the Two Untreated (Control) iPads

As mentioned above and shown in Figure 2, two tablet PCs were sampled once again without any additional reprocessing step in order to check for a potential germ-reducing effect due to the first sampling process itself. Re-sampling revealed reduction rates of 11.4% (228 of 246 CFU) on the nondisinfected iPad from the clinical setting and 22.4% (595 of 767 CFU) on the nondisinfected iPad from nonclinical setting only.

### Determination of the Quality of Cleaning With a Soft, Lint-Free Cloth Without Liquid Cleaning Agents

As already noted (see Figure 2), one tablet PC from the nonclinical study was cleaned only with a brand-new fleece according to the instructions of the manufacturer. The initial CFU count of this device at arrival in the laboratory was 891 CFU; 427 CFU remained after cleaning with a fleece (reduction rate: 51.1%). Removal of bacteria was rather higher for the glass surface of the front (231 of 234 CFU; 98.7% reduction), but almost no reduction (5 of 77 CFU; reduction rate: 6.5%) was achieved on the plastic part of the device. Cleaning the aluminium resulted in a CFU reduction from 580 to 352 corresponding to 38.3%.

**Table 1:** Recovery of pathogens found on the devices’ surfaces on initial arrival at the laboratory (shown as cumulative number of CFU from 10 tablet PCs each). Comparison of the total number of microorganisms: Mann-Whitney U test, *z=-*3.402; **P*=.*000670.

		Clinical setting			Nonclinical setting		
		Total CFU	Median CFU	IQR	Total CFU	Median CFU	IQR
**Total**		1842	162	125.75	4969	440	273.75
**Gram positive bacteria**		1825	160.5	122.75	4916	437.5	283
	Front (glass)	772	58.5	62.25	1,672	167	104.25
	Back (plastic)	214	22.5	27	481	46	35.5
	Back (aluminium)	839	63	68	2763	300.5	183.25
**Gram negative bacteria**		9	1	0.75	52	2	4.5
	Front (glass)	6	1	1	17	0.5	3.75
	Back (plastic)	0	0	0	5	0	1
	Back (aluminium)	3	0	0.75	30	0	1
**Other**		8	0	1.5	1	0	0
	Front (glass)	6	0	1.5	1	0	0
	Back (plastic)	1	0	0	0	0	0
	Back (aluminium)	1	0	0	0	0	0

**Table 2:** Distribution of species of microorganisms from the surface of the iPads on initial arrival of the devices at the laboratory (shown as cumulative number of colony forming units from 20 tablet PCs; n=6811).

		CFU	%	Gram stain
**Physiological human skin flora**				
	*Staphylococcus epidermidis*	1783	26.2	positive
	*Micrococcus luteus*	1509	22.2	positive
	*Staphylococcus hominis*	1256	18.4	positive
	*Staphylococcus capitis*	977	14.3	positive
	*Staphylococcus warneri*	194	2.9	positive
	Other coagulase-negative staphylococci	363	5.3	positive
	*Bacillus* spp*.*	309	4.5	positive
	*Corynebacterium* spp.	117	1.7	positive
	Other species	20	0.3	positive
**Pathogenic microorganisms**				
	*Staphylococcus aureus* (non-MRSA^a^)	218	3.2	positive
	*Pseudomonas* spp.	36	0.5	negative
	*Aspergillus* spp. / molds	9	0.1	N/A
	*Acinetobacter* spp*.*	8	0.1	negative
	Other species	12	0.2	negative

^a^MRSA: methicillin resistant *Staphylococcus aureus*.

**Table 3:** Reduction of bacteria on the surface of 6 iPads after standard disinfection procedure stratified by the type of previous usage (clinical vs nonclinical), the sample location (front vs back), the type of material (glass vs aluminium vs plastic), and type of Gram stain (positive vs negative).

		On laboratory arrival			After standardized disinfection			
		Total CFU	Median CFU	IQR	Total CFU	Median CFU	IQR	CFU reduction, %
**Total (clinical setting)**		753	121	65.75	14	2	1.5	98.1
**Gram positive bacteria**		749	121	65	14	2	1.5	97.2
	Front (glass)	280	39.5	42.75	5	1	0.75	96.9
	Back (plastic)	131	18	32.75	1	0	0	98.5
	Back (aluminium)	338	51	37.25	8	0.5	1.75	96.5
**Gram negative bacteria**		4	0.5	1	0	0	0	100.0
	Front (glass)	2	0	0.75	0	0	0	100.0
	Back (plastic)	0	0	0	0	0	0	100.0
	Back (aluminium)	2	0	0.75	0	0	0	100.0
**Total (nonclinical setting)**		2751	440	81	15	1	4.25	99.4
**Gram positive bacteria**		2739	437.5	78.25	15	1	5.25	99.5
	Front (glass)	816	148.5	107.5	10	1	2.75	97.9
	Back (plastic)	315	56.5	32	0	0	0	100.0
	Back (aluminium)	1608	300.5	90	5	0	0	99.7
**Gram negative bacteria**		12	1.5	3.25	0	0	0	100.0
	Front (glass)	8	0.5	2.5	0	0	0	100.0
	Back (plastic)	1	0	0	0	0	0	100.0
	Back (aluminium)	3	0	0.75	0	0	0	100.0

## Discussion

### Principal Findings

Without any doubt, mobile devices provide numerous advantages in a hospital setting, but despite these benefits, the potential risk of pathogen transmission must be taken into account [10]. There are several conclusions that can be drawn from the set-up and from the findings of our study.

As has been shown for other mobile devices [8,9], an extensive surface contamination also takes place when iPads are being used. Every fingerprint on the surface (Figure 1) will leave residue on the glass, aluminum, and plastic parts of the device (Figure 1) and may contain a large number of bacteria. An increased awareness of this fact is required when those devices are used during patient care. Brady et al [26] questioned 90 HCW (surgeons, anesthesiologists, and medical students) regarding this issue. At least 53% of them carried one mobile device (16% carried even more than one) including PDAs, mobile phones, and pagers. When asked about their cleaning habits, the HCW admitted that 80% of the PDAs, 85% of the mobile phones, and 96% of the pagers had never been cleaned by the owner.

Most of the pathogens are members of the resident or transient flora of its user (skin and/or anterior nose). Whatever microorganisms are present on the hands will be found on the mobile phone [27,28] or the tablet PC later on. This stresses the need for proper hand hygiene of HCW as it has been addressed by the World Health Organization in the international “clean hands campaigns” recently [29]. Patients, too, should be educated about the role of their own mobile devices brought to the hospital because these devices will also become contaminated [30]. Especially patients who harbor multidrug resistant bacteria should be discouraged to share their mobile phone with others.

As shown by the repeated sampling by contact plates described above, microorganisms may easily spread from the surface of the tablet PC when touched again. A transmission of pathogens that have caused nosocomial outbreaks has been shown for mobile phones [8,12]. One would assume that a much larger device such as a tablet PC is even more likely to serve as a vehicle of infectious agents. HCW should therefore be encouraged to perform alcohol-based hand rubs after using their mobile device [31].

Cleaning with a fleece as recommended by the manufacturer of the tablet PC showed a reduction of about 50% of microorganisms. However, a sufficient reduction of the microbiological load will be achieved only when proper disinfection is performed. A cleaning phase for visible contamination and a disinfection phase as a final decontamination step are considered most effective according to infection control as recommended in the guideline for environmental cleaning in health care facilities from the Centers for Disease Control and Prevention and the Healthcare Infection Control Practices Advisory Committee [3].

As has been shown for the disinfection of mobile phones [32], a disinfection procedure for iPads that makes use of isopropanol wipes is very effective in reducing and inactivating residual bacteria. However, one has to keep in mind that this procedure may cause a loss of the warranty for this product. It is noteworthy that Apple’s recommendations for the cleaning process of the iPad, available on the company’s website, have significantly changed in the past. The version from December 15, 2010, stated that “it is also safe to use isopropyl alcohol 70% or a similar product” for this purpose. However, in the meantime, this statement has been withdrawn. Instead, it is now specified that “liquid damage is not covered under the Apple product warranty or AppleCare Protection Plans” and specifically to “Avoid getting moisture in openings. Don’t use window cleaners, household cleaners, aerosol sprays, solvents, alcohol, ammonia, or abrasives to clean the iPad” [20].

Regular disinfection serves to maintain a significantly lower load of pathogens. Our study results imply that disinfection followed by the deBac-app has the ability to reduce microbiological flora in a quality manner. We would like to recommend using a standardized scheme for the disinfection process as described and controlled by the deBac application. Reprocessing of tablet PCs should be performed at least once a day, preferably at the beginning of the working shift. Additional courses of disinfection should also be carried out any time that visible contamination has occurred. Furthermore, we recommend disinfecting the device after using it in a patient room under isolation precautions (eg, if the patient harbors some type of multidrug resistant organism). The guided disinfection procedure ensures that all parts of the surface get thoroughly treated. Furthermore, all steps of reprocessing are documented and may be filed in infection control records.

### Limitations

Regarding the study design, we were willing to accept the following limitations: in accordance with our in-house regulations, machinery used in a clinical environment has to be disinfected. Therefore, it was impossible to learn about the baseline colonization by installing a control group on the wards and allowing this control group to use the devices without any disinfection. A randomized controlled trial or a controlled design with carefully matched comparison groups using standard practices as compared to the deBac condition would allow verification of the assumption that, when using mobile devices such as iPads in a clinical environment, performing an app-based disinfection process is more effective in reducing microbiological flora than simply using regular hand hygiene. This will have to be addressed in forthcoming studies.

The nursing staff was not provided with additional, paper-bound cleaning instructions since we wanted users to refer to the information available on the devices. We also refrained from collecting any personal data from the devices since our in-house data protection policy had to be followed. Only the information available from the anonymous cleaning protocols acquired from within the app was used for the evaluation and process documentation. The entries found in the log files demonstrated daily usage, but it is unknown to the observers how often and in which way the machines were used when the deBac-app was not running. This, of course, may bias the amount of CFU that were found on the surfaces.

As for the sensitivity and specificity of the various microbiological tests conducted, all laboratory methods that were used during the course of the study, including all microbiological tests for identification of pathogens, have been certified according to national guidelines. However, the sensitivity and specificity of taking the samples remains unclear as there is no so-called “gold standard” to compare with. It is known from optimized protocols that recovery may come up with a sensitivity of 98% and a specificity for a particular pathogen of interest of 95% [33]. However, standard environmental sampling still remains an unsolved problem these days [34,35]. Unfortunately, it is impossible compare the *Staphylococcus aureus* strains cultured from the nasal swabs of the users with those found on the devices themselves since the isolates had been discarded in the meantime. However, to us, it seems highly probable that we found corresponding strains here, as *Staphylococcus aureus* does not represent a typical “environmental” bacterium. Furthermore, it is well known that people tend to frequently touch their noses.

### Conclusions

Cleaning the devices with disinfecting wipes can be considered efficient and effective. Nevertheless, one must be aware of the potential danger of damaging the devices: there will definitely be a breach in warranty if liquid seeps into the device in any way and causes damage. On the other hand, although tablet PCs were originally developed for the consumer market, once they are used in the medical field, standardized methods for their disinfection must be implemented and closely followed. Also, manufacturers should become aware of the needs of the medical community regarding such devices. Thus, they might avoid building devices that—while being alluring for the medical sector—do not respect the demands for hygiene required for medicinal products. However, the most efficient personal action one can take to avoid transmission of bacteria, viruses, and other pathogens remains the proper disinfection of the hands before and after every patient interaction—this is a fact independent of the kind of device or any operating system or stated purpose.

Future studies should also take the specific profession of the staff as well as their level in the hierarchy into account. Their attitude towards using the deBac-app–based procedure compared to regular hand hygiene using alcohol-based disinfection solutions should also be evaluated. Also, the expenditure of time for implementing the procedure and other cost implicating variables will need to be addressed as the gathered results would be important factors for decision makers.

## Multimedia Appendix 1

Standardized disinfection process of an iPad as guided by the corresponding application (“deBac-app”), the app documents the cleaning attempts of the device’s frame, front and back side.

## Abbreviations

CFU: colony forming units
HCW: health care workers
IQR: interquartile range
MRSA: methicillin resistant Staphylococcus aureus
PDA: personal digital assistant
PLRI: Peter L Reichertz Institute for Medical Informatics
