# Supplementary material for: Standardized, App-Based Disinfection of iPads in a Clinical and Nonclinical Setting: Comparative Analysis
Source: J Med Internet Res. 2013 Aug 14;15(8):e176. doi: 10.2196/jmir.2643 (PMC3758047; doi:10.2196/jmir.2643)
Supplement: Supplementary file 1 [file jmir_v15i8e176_app1.pdf]

Multimedia Appendix 1. Standardized disinfection process of an iPad as guided by the corresponding application (“deBac-app”), the app documents the cleaning attempts of the device’s frame, front and back side.

Step 1	Step 2	Step 3	Step 4	Step 5	Step 6
Unplug all connectors from the device (for electricity safety reasons). Remove visible stains.	Use chemical resistant gloves (request due to work protection reasons). Use isopropanol wipes.	Wipe front until it has turned completely blue. Do not let liquids seep into the openings.	Wipe top of the frame. Proceed rotating in 90 degree steps until all sides of the frame have been cleaned.	Turn the device. Wipe the complete backside. This step has to take at least 10 seconds.	Check for leftover stains and repeat cleaning if necessary. Date and time will be documented.
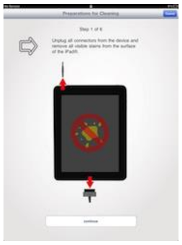	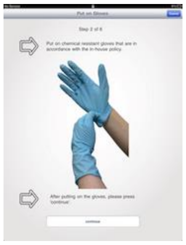	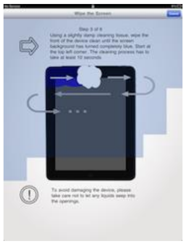	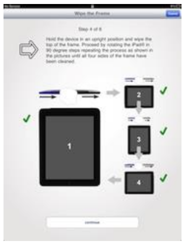	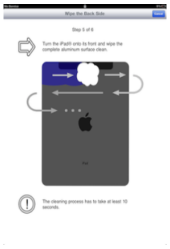	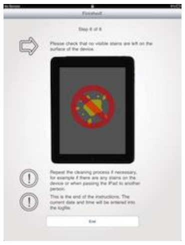
